# Re-Audit of Preadmission Handover Meeting on a Medium Secure Rehabilitation Ward

**DOI:** 10.1192/bjo.2025.10446

**Published:** 2025-06-20

**Authors:** Neeti Sud, Yousra Ghandour, Melissa Harris, Thomas Cranshaw

**Affiliations:** Cumbria Tyne and Wear NHS Foundation Trust, Morpeth, United Kingdom

## Abstract

**Aims:** Patients are admitted to our medium secure rehabilitation ward from high secure hospitals or other medium secure wards from within and outside our trust. We have a waiting list. There is extensive documentation and updates shared prior to any transfer over months. For patients within our trust, we share the same electronic records system. The legal status of most patients requires mandatory information sharing prior to any transfer for example via Ministry of Justice applications.

We examined our process of preadmission handover meetings for all five admissions in 2023–2024. We identified a lack of structured approach to preparing for the preadmission meeting. We concluded that a structured checklist may help. At the time of the re-audit in January 2025, we had two vacant beds and therefore two planned admissions from our waiting list were imminent in the coming weeks.

**Methods:** We used the following broad national, forensic and trust standards. NICE NG53: “1.2.7 During admission planning, record a full history or update that covers the person’s cognitive, physical and mental health needs, includes details of their current medication, identifies the services involved in their care.” Trust Policy: “Lead professional should make contact with service that covers the area the service user is to move to/from and arrange a formal hand over.” QNFMHS: “When patients are transferred between services there is a handover which ensures that the new team have an up to date care plan and risk assessment.”

We re-audited our service using a preadmission checklist based on last year’s audit to review what information has already been handed over and what needs to be specifically requested prior to admission. We then compared the preadmission meeting minutes of the last five admissions of 2023–2024 with the first two admissions of 2025 to reflect on our learning.

**Results:** There was no difference in terms of overall information sought by our team both pre- and post-audit. Updates were needed regarding physical and mental health and third party safeguarding information in the meeting.

**Conclusion:** Going through the preadmission list in preparation for the formal transfer meeting in a structured manner ensured any information gaps were identified prior to the preadmission meeting and timely requests made. There were reflections on the relational aspect of the information sharing process.

